# Contribution of Amino Acids in Cereal Grain Products to Precision Feed Formulation for Tambaqui (*Colossoma macropomum*) Using a Digestibility Approach

**DOI:** 10.1111/jpn.70000

**Published:** 2025-07-09

**Authors:** Danilo C. Proença, Monaliza F. Sena, Janaína G. Araújo, Guilherme W. Bueno, Igo G. Guimarães

**Affiliations:** ^1^ School of Agricultural Sciences, Department of Fisheries Resources and Aquaculture São Paulo State University (UNESP) Registro Brazil; ^2^ Laboratório de Pesquisa em Aquicultura Universidade Federal de Jataí Jataí Brazil; ^3^ Aquaculture Center of UNESP Jaboticabal Brazil

**Keywords:** amino acids, digestibility, energy, nutrients, nutritional value, tambaqui

## Abstract

Although cereal grain products have low protein content, they can contribute up to 25% of total protein in low‐trophic level/omnivorous fish diets. Thus, understanding the amino acid contribution of these ingredients becomes crucial for meeting the dietary requirements of omnivorous fish. A digestible nutrient basis is essential for precise feed formulation. To address this, we conducted a digestibility trial to assess cereal grain products' gross nutrient and amino acid digestibility for tambaqui, a key species in Latin American aquaculture. Forty‐eight tambaquis (400 ± 20 g) were randomly assigned to six 310 L aquaria connected to a recirculating system following a completely randomised block design with five treatments and four replicates. The fish were fed test diets consisting of a 70:30 mixture of the reference diet and test ingredients. Chromic oxide was used in all diets as the indigestible marker at 2 g kg^−1^. Results were analysed using ANOVA, and significant differences (*p* < 0.05) were further assessed using the SNK multiple range test. Our findings revealed that corn and sorghum exhibited higher levels of amino acids and digestible energy than other ingredients. At the same time, broken rice showed the lowest amino acid digestibility and wheat middlings had the lowest digestible energy content. Overall, protein and amino acid digestibility in the feed ingredients ranged from 70% to 99%. These results indicate that tambaqui can efficiently digest plant proteins, even when sourced from carbohydrate‐rich ingredients, underscoring the potential of these products in formulating cost‐effective diets for this species.

## 1 Introduction

Cereal grain products are carbohydrate‐rich ingredients and represent a significant component of fish feeds, primarily valued for their cost‐effectiveness as energy‐yielding substrates (Hertrampf and Piedad‐Pascual 2000; Guimarães et al. 2011; National Research Council 2011). These ingredients are crucial in formulating diets for fish species with diverse feeding habits. However, the ability to utilise dietary carbohydrates varies greatly among fish species. It is related to the feeding habits of the fish, molecular complexity and the feed processing technology they are submitted to (National Research Council 2011). Usually, carnivorous fish have limited ability to use carbohydrates as an energy source. Hence, their diets should not exceed 20% carbohydrate content, with variations depending on the carbohydrate source and feed processing technology. In contrast, omnivorous and herbivorous species demonstrate greater acceptance of diets containing carbohydrate levels up to 35%, and, similarly, this is dependent on the carbohydrate source and feed processing (Hertrampf and Piedad‐Pascual 2000).

The carbohydrate content varies significantly among cereal grain products and by‐products. For example, corn and rice typically contain around 80% of total carbohydrates, whereas wheat middlings (WM) and rice polishings (RP) have around 55% of total carbohydrates. Despite the prevalence of corn and sorghum, the cereal grain products most used in fish feeds are by‐products derived from the milling industry. Incorporating these by‐products into the fish feeds varies according to the feeding habits of the target species. Consequently, due to their moderate inclusion levels in omnivorous fish diets, these ingredients have been tested across several species to determine optimal inclusion levels and digestible energy (DE) content. This approach is fundamental for formulating cost‐effective and environmentally friendly diets (Gaylord and Gatlin 1996; Fagbenro 1999; Hertrampf and Piedad‐Pascual 2000; Rawles and Gatlin 2000; Tachibana et al. 2010; Vidal et al. 2017; Bicudo et al. 2018).

While the literature on the subject is extensive, a notable gap exists in studies assessing the amino acid (AA) digestibility of cereal grain products most utilised in omnivorous fish aquaculture. Existing research is limited, with only a handful of studies focusing on species such as tilapia (Köprücü and Özdemir 2005; Guimarães et al. 2008; Vidal et al. 2017), carp (Fagbenro 1999) and pacu (Fernandes et al. 2007; Abimorad et al. 2008). Additional studies have explored digestibility in other species, such as rainbow trout (Magalhães et al. 2017) and dourado (*Salminus brasiliensis*) (Moro et al. 2017), providing valuable insights into the variability of nutrient utilisation across species with different feeding habits. However, none of these studies have precisely estimated the contribution of the AAs from these ingredients to meet the requirements of the studied species.

The rising costs of fishmeal and the increasing demand for sustainable aquaculture practices have driven significant interest in plant‐based ingredients for aquafeeds. Recent studies have highlighted the potential of plant‐derived ingredients, like cereal grains and their by‐products, to supply essential amino acids (EAAs) and energy for omnivorous fish species, such as tambaqui (Nascimento et al. 2020; Wangkahart et al. 2023). These ingredients are particularly relevant in the context of cost‐effective formulations aimed at large‐scale aquaculture, where balancing nutrient bioavailability and minimising environmental impacts are critical. While challenges like antinutritional factors persist, advances in processing techniques and the inclusion of enzyme supplements have shown promise in enhancing nutrient utilisation from plant‐based feeds (Wangkahart et al. 2023).

To build upon these findings and address the gaps identified, the digestibility approach provides a valuable framework for assessing nutrient bioavailability, enabling the formulation of diets tailored to aquaculture species' specific nutritional needs while promoting efficiency and sustainability (P et al. 2022). This method evaluates how efficiently animals absorb and excrete nutrients, offering insights into the bioavailability of feed components. By minimising nutrient waste, the digestibility approach supports precise feed formulation and helps reduce environmental impacts, making it essential for sustainable aquaculture practices. Using this method, we evaluated how efficiently tambaqui can utilise the protein and energy from cereal grains, contributing to cost‐effective production systems.

Therefore, this study conducted a digestibility trial to determine the coefficients of total tract apparent digestibility (CTTADs) of nutrients from the most used cereal grain products and by‐products in omnivorous fish feed formulation. Additionally, we estimated the contribution of these ingredients in meeting AA requirements, using tambaqui (*Colossoma macropomum*), the second most farmed neotropical fish in Latin America, as a model for omnivorous species.

## 2 Materials and Methods

The Animal Ethics Committee of the Universidade Federal de Goiás approved all the procedures performed in this study (protocol 149/10‐CEUA).

### 2.1 Diet Preparation

Diets were formulated using conventional feed ingredients commonly employed in omnivorous fish diets. Initially, a reference diet (RD) was formulated based on the available data on nutrient requirements for tambaqui (Guimarães and Martins 2015). The ingredients and chemical composition of the RD are provided in Tables 1 and 2, respectively. This study evaluated five cereal grain products and by‐products: WM, broken rice (BR), RP, sorghum, and ground corn meal.

**Table 1:** Composition and proximate analysis of reference diet.

Ingredient	g kg^−1^
Soybean meal	490.0
Fish meal	50.0
Corn	272.5
Wheat middlings	60.0
Soybean oil	65.0
DL‐Methionine	1.0
Dicalcium phosphate	56.0
Vit/min supplement^1^	2.5
Ascorbic acid^2^	0.8
NaCl^3^	1.0
Chromic oxide III	2.0
BHT^4^	0.2

^1^ Vitamin and mineral mix provided the following (IU or mg kg^−1^ diet): folic acid 10 mg, biotin 10 mg, niacin 170 mg, calcium pantothenate 80 mg, vitamin A 16,060 IU, vitamin B1 32 mg, vitamin B12 0.032 mg, vitamin B2 32 mg, vitamin B6 32 mg, vitamin C 350 mg, vitamin D3 4,510 IU, vitamin E 250 IU, vitamin K3 30 mg. Cobalt 0.5 mg, Copper 20 mg, Iron 150 mg, Iodine 1 mg, Manganese 50 mg, Selenium 0.7 mg and Zinc 150 mg, supplied by Mogiana Alimentos Ltd.

^2^ Ascorbyl polyphosphate (DSM Stay C 35.0% activity).

^3^ Sodium chloride.

^4^ Antioxidant: butylated hydroxytoluene.

The indirect method was employed for the digestibility evaluation, and the inert marker used was chromic oxide at a concentration of 2.0 g kg^−1^ of diet. Test diets containing the evaluated ingredients were prepared by replacing 30% of the RD with the test ingredient. The exceptions were the micronutrient mixture and the inert marker.

All the ingredients were ground to a 500 μm diameter, mechanically mixed, and then water (25% of dry weight) and oil were added to form a moisture mixture. Then, the diets were extruded using a single screw laboratory extruder with 1 mm die holes to produce pellets with approximately 3–4.0 mm diameter. The test diets were dried in a forced‐air oven at 55°C until the moisture content was below 10% (approximately 24 h), packaged in sealed plastic bags, and stored at −18°C until use.

### 2.2 Experimental Fish, Facilities and Faecal Collection Procedure

The experimental set‐up consisted of two sets of aquaria connected to a water‐recirculating system with a thermostat‐controlled, air blower and air stones. One set consisted of six 310 L aquaria, each with a diameter of 90 cm, which were used for the feeding procedure. In contrast, the other set consisted of three 200 L conic‐bottomed aquaria used for faecal collection. Water quality parameters, including pH, dissolved oxygen, ammonia and nitrite, were monitored throughout the experiment and remained within the optimum range for raising tropical fish species (Baldisserotto and Gomes 2020). A multi‐parameter probe (Akso, AK88) was used for pH and dissolved oxygen measurements, while commercial colorimetric kits were employed for ammonia and nitrite analysis.

We employed a faecal collection by sedimentation following a modified procedure of the Guelph system, which was described previously by Guimarães et al. (2008) and adapted to collect tambaqui faeces (da Mota et al. 2015). This method reduces nutrient leaching in tambaqui faeces when utilising the sedimentation method of faecal collection. Forty‐eight tambaquis (400 ± 20 g) were randomly distributed among the six 310 L aquaria, forming six groups of eight fish each. These aquaria were exclusively used for the feeding procedure to prevent faecal contamination from the feed. Then, diets (comprising five test diets and the RD) were randomly assigned to the aquaria, and the fish groups were fed their respective diets for 7 days. On the 8th day, three fish groups were randomly selected and transferred to the conic‐bottomed aquaria designated for faecal collection, where they remained for 8 h before returning to their respective feeding aquaria. Faeces were collected at 30‐min intervals in a chilled bottle connected to the bottom of the aquaria. On the 9th day, the remaining three fish groups were transferred to the faecal collection aquaria, and the same procedure was repeated. This procedure was conducted three times for each fish group. As described below, replication was defined as the pooled faeces collected from each fish group during each round.

The diets were then reassigned to the tanks, and a new faecal collection was conducted after a 7‐day adaptation period to the diets. Each faecal collection period with the assigned diets was regarded as one round. In total, we performed four rounds of faecal collection for each diet. Although the same fish groups were used across rounds, this approach allowed for repeated measures of faecal output over time. Therefore, these repetitions were considered temporal replicates, as commonly adopted in fish digestibility trials with limited faecal output per day (see Araújo et al. 2018). The fish were fed twice daily throughout the experiment.

### 2.3 Calculations

The CTTAD for the test diets was calculated according to the equation described by Cho (1993):

$${\mathrm{CTTAD}}_{\left(n\right)}=100\left[100\left(\frac{{\%\mathrm{Cr}2O3}_{d}}{{\%\mathrm{Cr}2O3}_{f}}\right)\times \left(\frac{{\%N}_{f}}{{\%N}_{d}}\right)\right]$$

Where CTTAD(*n*) = the CTTAD of a nutrient or energy in the test diets, Cr_2_O_3_d = % chromic oxide of the diet, Cr_2_O_3_f = % chromic oxide of the faeces, *N_d_* = nutrient in the test diet and *N_f_* = nutrient in faeces.

The CTTAD of nutrients from each ingredient was calculated according to the equation proposed by Bureau and Hua (2006):

$${\mathrm{CTTADN}}_{\mathrm{ing}}=\frac{\left[\left(a+b\right)\times {\mathrm{CTTADN}}_{\mathrm{test}}-a\times {\mathrm{CTTADN}}_{\mathrm{ref}}\right]}{b}$$

Where a = nutrient contribution of the RD to the nutrient content of the test diet, b = nutrient contribution of test ingredients to the nutrient content of the combined diet, a + b = level of nutrient in the combined diet (%), CTTADN_test_ = CTTAD of a nutrient in the combined diet and CTTADN_ref_ = CTTAD of a nutrient in the RD.

The digestible nutrient content of the ingredients was calculated by multiplying the analysed nutrient content in feed ingredients by the respective CTTAD value. The AA contribution was estimated based on the AA requirement for tambaqui as assessed in Martins et al. (2024). Ingredient inclusion recommendations for corn middlings and WM, the most used cereal grain products in omnivorous fish diets, were based on reported guidelines (Hertrampf and Piedad‐Pascual 2000). The carbohydrate ratio was also maintained under the limit established for omnivorous fish (20%–40%) (Hertrampf and Piedad‐Pascual 2000; National Research Council 2011).

### 2.4 Chemical Analysis

The chemical composition, including dry matter (DM), ash, crude protein (CP), crude fibre and total lipid content, was determined according to standard methods outlined in Helrich and the Association of Official Analytical Chemists. According to Bremer Neto et al. (2005), chromic oxide was determined using a colorimetric method, and gross energy (GE) content was measured using a calorimeter (Parr Instrument Company, Moline, Illinois, the United States). AA analysis was conducted using high‐performance liquid chromatography following acid and base digestion, using an ion exchange chromatographic analysis method. After digestion, solutions of the free AA pools were vacuum filtered, diluted to 0.25 M with HCl 0.02 N for adjustment to pH 8.5, and filtered through a Millipore membrane (0.45 mm). AAs were quantified photocolorimetrically after staining with Ninhydrin (Sindirações 2009).

### 2.5 Statistics

The RD was evaluated throughout the trial, but it was only used to calculate the CTTADs. Therefore, the data was not included in the statistical analysis. The trial consisted of five treatments (test diets/ingredients) and four replicates (groups of fish) arranged in a randomised block design for the CTTAD of DM, CP and GE. Due to the limited samples from the last faeces collection, three replicates were used for the CTTAD of individual AAs. The faecal collection rounds were blocked in the statistical analysis to account for potential variations due to the faecal collection period. The differences between ingredients were analysed according to the following model:

$$Y_{\mathrm{ijk}}=µ+T_{i}+B_{j}+e_{\mathrm{ijk}}$$

Where *Y* is the observed response, *µ* is the overall mean, T is the effect of ingredients, B is the effect of the faecal collection period and *e* is the residual error.

All percentage data were arcsine transformed before analysis. The CTTAD values for DM, protein, energy and individual AAs were subjected to ANOVA using the PROC GLM procedure of SAS (Statistical Analysis System, version 8.12). Differences in CTTAD values between the tested ingredients were determined using the Student–Newman–Keuls multiple range test at *p* < 0.05. No effect of the faecal collection period (round) was observed (*p* = 0.1093). Additionally, the CTTAD data of all test diets were analysed by linear correlation using the PROC CORR procedure of SAS to investigate nutrient interactions. Since the interactions among the CTTAD of AAs were not significant, we removed these data from the publication.

## 3 Results

All the cereal grain products evaluated in this study showed similar DM content, with slight differences in GE, except for the RP, which showed the highest GE content (Table 2). Rice by‐products showed the highest protein content among the feed ingredients, while corn and sorghum exhibited the lowest values. The fibre content varied significantly among the cereal products, ranging from 3.2 to 70.5 g kg^−1^. Although the EAA composition varied considerably among the feed ingredients, most variations did not exceed 50%, except for Arg and Lys in RP and WM, which were highest among all the cereal grain products.

**Table 2:** Chemical and amino acid composition (in g kg^−^^1^) and energy content (in MJ kg^−^^1^) of cereal grain products and the reference diet (dry matter basis).

Nutrients	BR^1^	Corn	RP^2^	Sorghum	WM^3^	RD^4^
IFN	5‐28‐242	4‐02‐935	4‐03‐943	4‐04‐444	4‐05‐205	
Dry matter	889.4	886.5	903.6	901	894	938.5
Crude protein	155.2	85.9	138.5	93	118.9	287
Gross energy	17.0	16.3	21.5	16.7	18.0	18.5
Fibre	3.2	17.5	46.3	16.9	70.5	55.4
Lipid	1.0	36.2	146.4	19.2	27.5	81.1
N‐free extract	711.8	735.7	514.1	759.6	631.6	421.1
Ash	6.0	11.2	58.3	12.3	45.5	93.9
Amino acids						
Essential						
Arg	5.6	3	9.2	2.8	9.1	17.0
His	1.7	2	3.2	1.9	3.5	6.7
Ile	3.1	2.4	4.1	2.3	4.5	11.9
Leu	6	7.7	8.1	7.7	8.4	21.7
Lys	2.7	2.1	6.4	2.1	6.2	14.0
Phe	4.4	3.8	5.8	3.7	6.3	13.6
Met	1.1	0.9	0.8	1	0.9	4.7
Thr	2.8	2.6	4.9	2.6	4.8	10.8
Val	4.5	3.3	6.5	3.3	6.7	12.7
Non‐essential						
Ala	4.2	4.9	7.7	4.9	7.1	13.3
Asp	7.2	4.7	11.9	4.7	10.7	29.8
Cys	0.7	0.8	1.4	0.9	1.6	3.4
Gly	3.6	2.8	6.7	2.8	7.8	14.4
Glu	14.4	12.8	18.1	13.4	28.8	51.1
Tyr	1.5	1.2	2.5	1.2	2.8	8.6
Ser	3.8	3.2	5.4	3.2	6	14.5

^1^ Broken rice.

^2^ Rice polishings.

^3^ Wheat middlings.

^4^ Reference diet.

Sorghum showed the highest CTTAD values for DM and GE, while WM had the lowest values. However, no differences were observed among sorghum and the other cereal grain products evaluated. The CTTAD of CP was more consistent among feed ingredients and did not vary significantly (Table 3). The highest DE was recorded for sorghum, while BR, corn and RP had moderately high DE values. The lowest DE was recorded for WM (Table 3).

**Table 3:** Coefficients of total tract apparent digestibility (CTTADs) of dry matter (DM), protein (CP), and energy (GE) in cereal grain products and by‐products for tambaqui (mean ± SD, *n* = 4).

Feed ingredients	Apparent digestibility coefficient			Digestible energy (MJ kg^−1^)
	DM	CP	GE	
Rice polishings	0.734 ± 0.03^b^	0.958 ± 0.02^ab^	0.785 ± 0.03^b^	15.25^b^
Wheat middlings	0.575 ± 0.04^c^	0.930 ± 0.03^a^	0.511 ± 0.03^c^	9.25^c^
Corn	0.915 ± 0.03^a^	0.962 ± 0.01^a^	0.888 ± 0.03^a^	16.09^a^
Broken rice	0.918 ± 0.03^a^	0.899 ± 0.02^b^	0.891 ± 0.02^a^	15.92^a^
Sorghum	0.972 ± 0.01^a^	0.968 ± 0.02^a^	0.962 ± 0.02^a^	17.69^a^

*Note:* Different superscript letters in the columns differ by SNK test (*p* < 0.05).

The nutrient content of test diets had varying effects on the CTTAD of nutrients from cereal grain products for tambaqui (Table 4). Overall, increased fibre, CP and ash in test diets negatively affected the CTTAD of DM and GE (*p* < 0.01). However, no significant effect of any of these compounds on CTTAD of CP in cereal grain products was observed.

**Table 4:** Correlation coefficients between nutrient content and CTTAD of nutrients in test diets containing different cereal grain products.

Nutrients	CTTAD DM	CTTAD CP	CTTAD GE
Fibre	−0.93**	0.10	−0.90**
GE	−0.24	0.38	−0.02
CP	−0.84**	0.16	−0.79**
Ash	−0.97**	−0.01	−0.93**

*Note:* Each value represents a correlation coefficient between nutrient concentration in the test diet and digestibility.

** *p* < 0.01.

The digestibility of EAAs showed a narrow variation among the feed ingredients (Table 5). EAA digestibility generally exceeded 85%, except for Met in BR, which demonstrated the lowest digestibility among all EAAs of the feed ingredients (70.6%). Although a similarly high digestibility of non‐essential amino acids (NEAAs) was observed, Ala and Cys showed the lowest digestibility in BR (*p* < 0.01). In general, the digestibility of the other NEAAs was higher than 83%. The mean AA digestibility of each ingredient correlated well with the CTTAD of CP in all cereal grain products, with correlation coefficients exceeding 0.87 (*p* < 0.01).

**Table 5:** Coefficients of total tract apparent digestibility of amino acids from cereal grain products for tambaqui (*n* = 3).

EAA	BR^3^	Corn	RP^4^	Sorghum	WM^5^
Arg	0.895 ± 0.02^b^	0.973 ± 0.02^a^	0.904 ± 0.05^b^	0.874 ± 0.03^a^	0.901 ± 0.04^b^
His	0.956 ± 0.04^b^	0.977 ± 0.01^a^	0.945 ± 0.04^ab^	0.992 ± 0.01^a^	0.919 ± 0.03^b^
Ile	0.926 ± 0.03^b^	0.992 ± 0.01^a^	0.951 ± 0.03^ab^	0.986 ± 0.01^ab^	0.921 ± 0.03^ab^
Leu	0.907 ± 0.03^b^	0.993 ± 0.01^a^	0.928 ± 0.04^b^	0.969 ± 0.02^b^	0.924 ± 0.03^b^
Lys	0.959 ± 0.02^b^	0.991 ± 0.01^a^	0.944 ± 0.02^bc^	0.993 ± 0.01^a^	0.926 ± 0.01^c^
Phe	0.866 ± 0.03^b^	0.991 ± 0.01^a^	0.898 ± 0.03^b^	0.877 ± 0.03^ab^	0.913 ± 0.04^b^
Met	0.706 ± 0.03^c^	0.986 ± 0.02^a^	0.884 ± 0.03^b^	0.979 ± 0.02^a^	0.947 ± 0.02^a^
Thr	0.890 ± 0.02^b^	0.976 ± 0.02^a^	0.899 ± 0.02^b^	0.971 ± 0.01^a^	0.904 ± 0.01^b^
Val	0.891 ± 0.03^b^	0.992 ± 0.01^a^	0.926 ± 0.03^b^	0.972 ± 0.02^a^	0.959 ± 0.02^b^
Mean EAA	0.888	0.986	0.920	0.957	0.924
NEAA					
Ala	0.731 ± 0.03^b^	0.988 ± 0.02^a^	.842 ± 0.02^ab^	0.968 ± 0.02^b^	0.893 ± 0.02^ab^
Asp^1^	0.926 ± 0.02^a^	0.994 ± 0.01^a^	0.948 ± 0.02^a^	0.987 ± 0.01^a^	0.957 ± 0.02^a^
Cys	0.761 ± 0.02^c^	0.983 ± 0.02^a^	0.832 ± 0.02^b^	0.964 ± 0.03^a^	0.828 ± 0.02^b^
Gly	0.901 ± 0.03^a^	0.994 ± 0.01^a^	0.952 ± 0.03^a^	0.938 ± 0.02^a^	0.973 ± 0.02^a^
Glu^2^	0.952 ± 0.04^b^	0.993 ± 0.01^a^	0.937 ± 0.02^a^	0.992 ± 0.01^ab^	0.947 ± 0.03^a^
Tyr	0.893 ± 0.03^c^	0.994 ± 0.01^a^	0.930 ± 0.03^bc^	0.972 ± 0.02^ab^	0.928 ± 0.03^ab^
Ser	0.886 ± 0.03^b^	0.990 ± 0.01^a^	0.886 ± 0.02^ab^	0.944 ± 0.02^a^	0.917 ± 0.03^ab^
Mean NEAA	0.864	0.991	0.904	0.966	0.920
Mean AA	0.876	0.989	0.912	0.962	0.922
CTTAD CP	0.899	0.962	0.958	0.968	0.930

*Note:* Means ± SD with different superscript letters in the same column are statistically different by SNK test (*p* < 0.05).

^1^ Aspartic acid + asparagine.

^2^ Glutamic acid + glutamine.

After calculating the digestible AA levels in the cereal grain products evaluated (Table 6), we observed that although WM and RP had relatively low digestibility coefficients for DM and energy, they were the richest cereal grain products in digestible Arg and Lys, two critical AAs in fish nutrition. However, the digestible Met content of the feed ingredients was quite similar.

**Table 6:** Digestible amino acid content of cereal grain products for tambaqui (g kg^−^^1^).

Amino acids	Corn	BR^1^	Sorghum	WM^2^	RP^3^
Essential					
Arg	2.9	5.0	2.5	8.2	8.3
His	2.0	1.6	1.9	3.2	3.0
Ile	2.4	2.9	2.3	4.1	3.9
Leu	7.6	5.4	7.5	7.8	7.5
Lys	2.1	2.6	2.1	5.7	6.0
Phe	3.8	3.8	3.2	5.8	5.2
Met	0.9	0.8	1.0	0.9	0.7
Thr	2.5	2.5	2.5	4.3	4.4
Val	3.3	4.0	3.2	6.4	6.0
Non‐essential					
Ala	4.8	3.1	4.7	6.3	6.5
Asp	4.7	6.7	4.6	10.2	11.3
Cys	0.8	0.5	0.9	1.3	1.2
Gly	2.8	3.2	2.6	7.6	6.4
Glu	12.7	13.7	13.3	27.3	17.0
Tyr	1.2	1.3	1.2	2.6	2.3
Ser	3.2	3.4	3.0	5.5	4.8
Sum	57.6	60.6	56.5	107.3	94.5
DP^4^	89.4	77.3	115.1	144.3	132.6

^1^ Broken rice.

^2^ Wheat middlings.

^3^ Rice polishings.

^4^ Digestible protein.

The contribution of AAs from corn meal and WM to meet the estimated AA requirements of tambaqui is detailed in Table 7. Phenylalanine presented the highest contribution, at 10.43% of the estimated requirement, followed by leucine (7.64%) and histidine (7.06%). Arginine and lysine showed the lowest contributions, meeting 4.10% and 4.44% of the requirements, respectively. These values reflect the calculated contributions of the tested cereal grain products based on their inclusion levels in the diet.

**Table 7:** Contribution of amino acids to meet the estimated amino acid requirement of tambaqui^a^.

Essential **amino acids**	Contribution^b^ (g kg^−1^)	Tambaqui^c^ (g kg^−1^)	% Contribution^d^
Arg	1.1	26.8	4.10
His	0.6	8.5	7.06
Ile	0.8	16.2	4.94
Leu	2.3	30.1	7.64
Lys	0.8	18.0	4.44
Phe	1.7	16.3	10.43
Met	0.5	10.3	4.85
Thr	0.9	17.5	5.14
Val	1.1	19.5	5.64

^a^ Calculated based on a diet with 300 g kg^−^^1^ protein.

^b^ Calculated based on a diet containing 250 g kg^−^^1^ of corn meal and 100 g kg^−^^1^ of wheat middlings.

^c^ Estimated amino acid requirement (Martins et al. 2024).

^d^ Percentage of the tambaqui requirement.

## 4 Discussion

While many studies have evaluated the CTTAD of gross nutrients from several cereal grain products in fish, few have specifically focused on the digestibility of AAs in these feed ingredients. In this study, we conducted a digestibility trial to comprehensively assess the CTTAD of nutrients from selected cereal grain products for tambaqui, using this species as a model to demonstrate the contribution of digestible AAs to omnivorous fish diets. Our findings offer valuable insights into the role that these AAs play in the nutrition of omnivorous fish species.

The nutrient composition of the ingredients in this study was similar to the database found in the literature (Hertrampf and Piedad‐Pascual 2000; National Research Council 2011). However, WM exhibited a lower protein and lipid content, while RP had a higher protein and lower N‐free extract content than the database. Differences in nutrient composition are expected to occur in plant products due to various factors that may affect the amount of gross nutrients. For example, soil fertility and milling procedures affect plant products' nutrient composition (Hertrampf and Piedad‐Pascual 2000; Glencross et al. 2007; Guimarães et al. 2008).

Generally, the CTTAD of GE and DM are positively correlated and vary significantly according to the fish species (Guimarães et al. 2008; Guimarães et al. 2011; National Research Council 2011). Omnivorous and herbivorous fish tend to efficiently use the DM in plant products rich in carbohydrates due to their enzymatic efficiency in digesting, absorbing and utilising carbohydrates (Cho 1993; Hertrampf and Piedad‐Pascual 2000; National Research Council 2011). Since DM mainly consists of the organic fraction of an ingredient, several studies use organic matter to estimate GE digestibility. Our results indicated that tambaqui is an omnivorous fish that efficiently uses carbohydrate‐rich plant products, as previously demonstrated through the quantification of the enzyme activity of the digestive tract (Corrêa et al. 2007). Indeed, we have observed that tambaqui showed higher CTTADs of GE and DM and DE values for corn, sorghum and BR compared to previously reported values for Nile tilapia (Pezzato et al. 2002; Furuya et al. 2004; Guimarães et al. 2008), a traditional omnivorous fish used in intensive aquaculture.

Although tambaqui efficiently utilises cereal grain products in their diets, an increase in fibre content seems to reduce their ability to digest these ingredients. This effect of fibre in fish is usually observed regardless of the feeding habit (Gaylord and Gatlin 1996; Rawles and Gatlin 2000; Abimorad et al. 2008; Bicudo et al. 2018). Nevertheless, previous results with tambaqui emphasise its efficiency in using dietary fibre (Lima and Goulding 1998; Baldisserotto and Gomes 2020), which contradicts our results. It is possible that tambaqui's digestive physiology resembles that of other fish species, where interactions between fibre and other compounds, such as lipids, enhance fibre digestibility (Guimarães et al. 2014). Additionally, while this study focused on total fibre content, the fibre composition (soluble vs. insoluble) may further influence nutrient absorption and digestibility efficiency in tambaqui. Future studies should explore these interactions and investigate the specific effects of fibre types to better understand the gut physiology mechanisms underlying tambaqui's potential for digesting different types of carbohydrates.

Our findings on the digestibility of cereal grain products in tambaqui align with studies conducted on other aquaculture species. For instance, juvenile pacu (*Piaractus brachypomus*) efficiently utilised selected feedstuffs, with digestibility coefficients influenced by ingredient processing and composition (Magalhães et al. 2017). Similarly, dourado (*S. brasiliensis*) showed that feed processing significantly impacts carbohydrate digestibility, highlighting the importance of ingredient‐specific evaluations (Moro et al. 2017). These results underscore the variability in nutrient utilisation among species and emphasise the need for tailored formulations based on the digestive physiology of each species. Moreover, studies on rainbow trout have explored the apparent digestibility of European grain legumes, providing valuable comparative data for understanding the role of plant‐based ingredients in fish nutrition (Magalhães et al. 2017).

Compared to the available database on feed ingredients (Hertrampf and Piedad‐Pascual 2000; National Research Council 2011), Met content appears to be lower in RB, while the AAs with lower content in sorghum were Arg, Ile, Leu and Val. WM's Ile and Leu content was 50% lower than the reported values for this ingredient (Hertrampf and Piedad‐Pascual 2000; National Research Council 2011). However, the other AAs seem to be in the range of the reported values in the ingredients. Although we have observed these effects on the AA composition, this seems not to interfere with the ability of this species to digest the protein fraction since no correlation was observed between the AA content and CTTAD of single or multiple AAs (data not shown).

The mean AA digestibility correlated well with the CTTAD of protein, indicating the reliability of the data provided in this study. Similar correlations have been previously reported for several fish species in different studies (Gaylord et al. 1996, 2004; Furuya et al. 2004; Abimorad et al. 2008; Dong et al. 2010; National Research Council 2011; Vidal et al. 2017). These observed differences in the AA composition may not have affected the CTTAD of protein and the digestibility of other AAs, as the amount of these AAs in these ingredients might not be sufficient to meet the requirements for any fish species.

Nonetheless, we calculated the contribution of the digestible AAs from the cereal grain products evaluated in this study to assess their potential to meet the AA requirement for tambaqui. Although the contribution rate was relatively low (around 6%), some AAs could contribute up to 10.5%, considering a diet containing 250 g kg^−1^ of corn and 100 g kg^−1^ of WM. However, it is essential to note that this contribution might vary depending on the limitation of carbohydrate inclusion. Additionally, despite some cereal grains having lower DE levels (i.e., WM and RP), they showed a higher content of digestible limiting AAs (Arg and Lys in RP and WM), indicating that these ingredients could assist in adjusting the digestible AA content in omnivorous fish feed formulation. It is worth noting that none of the cereal grains evaluated in this study, when considered individually, could fully meet the estimated EAA requirements for tambaqui. Thus, AA supplementation, particularly of lysine, methionine and threonine, may be necessary depending on the formulation matrix, especially when corn, sorghum or BR are used as the main carbohydrate sources.

## 5 Conclusion

In conclusion, our study demonstrated that sorghum, corn and BR are viable energy sources for formulating cost‐effective diets for tambaqui, potentially contributing significantly to meeting the species' nutritional requirements. Additionally, our study elucidated that these ingredients could contribute up to 13% of the digestible EAAs in an omnivorous fish diet, highlighting their significance in diet formulation for species like tambaqui.

These findings have practical implications for aquaculture, offering valuable insights into developing environmentally friendly and economically viable feed formulations. Aquafeed manufacturers can optimise diet formulations using locally available and cost‐effective ingredients while minimising production costs and environmental impact. Furthermore, future research directions may include investigating the synergistic effects of different dietary components, assessing the long‐term impacts of formulated diets on fish performance and expanding the study to other fish species to validate our findings. Additionally, if other species from the same phylogenetic group (i.e., *Piaractus mesopotamicus and P. brachypomus*) share similar physiological and nutritional characteristics, this information could facilitate the formulation of species‐specific diets. By addressing these aspects, we can further enhance our understanding of fish nutrition and contribute to the sustainability and efficiency of aquaculture practices.

## Ethics Statement

The authors confirm that the journal's ethical policies, as noted on the journal's author guidelines page, have been adhered to. All procedures performed in this study were approved by the Animal Ethics Committee of the Universidade Federal de Goiás (protocol number 149/10‐CEUA). The authors have ensured that the ethical standards for the use of animals in research, as recognised by the relevant Brazilian legislation, are consistent with international ethical practices.

## Data Availability

The data that support the findings of this study are available from the corresponding author upon reasonable request.
